# Wear resistance and color stability of innovate esthetical Bioflx crowns compared to zirconia pediatric crowns

**DOI:** 10.1186/s12903-025-05672-5

**Published:** 2025-03-15

**Authors:** Noha El-Sayed Fathi Abdou, Eman Mohamed Mohamady, Tarek Mohamed Nabil Mohamed Kamel Mahmoud, Asmaa Ali Emam Abo-Elsoud

**Affiliations:** 1 Pediatric Dentistry, Preventive Dentistry and Dental Public Health, Faculty of Dentistry, Aswan University, Aswan City, Egypt; 2 Dental Biomaterials Department, Faculty of Dentistry, Suez Canal University, Ismailia City, Egypt; 3 Pediatric Dentistry, Preventive Dentistry and Dental Public Health, Faculty of Dentistry, Suez Canal University, Ismailia City, Egypt; 4 Pediatric Dentistry, Preventive Dentistry and Dental Public Health, Faculty of Dentistry, Suez Canal University, Ismailia City, Egypt

**Keywords:** Zirconia crowns, Bioflx crowns, Wear, Color stability

## Abstract

**Background:**

Zirconia crowns are the most common aesthetic option for full coverage in pediatric dentistry. Bioflx crowns have been recently introduced, offering a unique combination of aesthetics, flexibility, and durability.

**Aim:**

The objective of this study was to evaluate and compare the wear of the crowns and opposing enamel. Additionally, to assess the color stability of Bioflx pediatric crowns following thermodynamic aging compared to zirconia crowns.

**Methods:**

Forty acrylic resin dies were fabricated based on specific criteria and equally divided into two groups: zirconia crowns and Bioflx crowns. Thermodynamic cycling was conducted to simulate oral conditions over six months. Wear resistance was quantitatively assessed utilizing a Universal Serial Bus (USB) digital microscope with an integrated camera. Color stability was measured using a spectrophotometer before and after thermal aging and following immersion in various solutions (water, milk chocolate, orange juice, and cola). Data were collected, tabulated, and statistically analyzed using the Shapiro–Wilk test and paired sample t-tests. The level of statistical significance was set at a *p*-value of 0.05.

**Results:**

A statistically significant difference was observed between zirconia and Bioflx crowns regarding volume loss and average roughness height [µm] of the opposing enamel (*P* = 0.021 and 0.001, respectively). Furthermore, there were significant differences in volume loss (µm^3^) and average roughness height (µm) between the zirconia and Bioflx crowns (*P* < 0.001). In contrast, the results of color change revealed non-significant differences between zirconia and Bioflx crowns (*P* = 0.470) before and after thermocycling aging, as well as following immersion in different solutions.

**Conclusions:**

Zirconia crowns cause more wear on opposing natural teeth than Bioflx crowns. Bioflx crowns show a higher average wear rate than zirconia. There are no significant differences between the two crown’s materials in terms of color change after aging and immersion in various solutions.

## Introduction

The American Academy of Pediatric Dentistry recommends full-coverage restorations in children with severely decayed, pulpotomized, damaged, or traumatized teeth [1]. Zirconia crowns are frequently used as tooth-colored restorative materials due to their favorable biological, mechanical, and aesthetic properties [2].

However, zirconia crowns pose several challenges. They require significant removal of tooth structure, potentially prolonging the duration of dental procedures. Manufacturers also advocate for passive seating of these crowns, primarily depending on dental cement for retention [3]. The dependence on cement, along with the challenges in modifying crown margins, presents clinical challenges. Furthermore, zirconia crowns may be associated with increased wear on opposing enamel. Finally, their high cost renders them a more costly aesthetic option than other materials [4].

A novel high-strength, tooth-colored crown, the NuSmile Bioflx crown, has been introduced to mitigate the limitations associated with zirconia crowns. This crown is made of biocompatible hybrid resin polymer materials that provide significant strength and flexibility [5]. Notably, it is BIS-GMA free, devoid of metals, and requires tooth preparation similar to stainless-steel crowns. Bioflx crowns demonstrate superior wear resistance compared to stainless steel crowns and can self-adapt by forming a dimple in areas of high occlusion [6]. These crowns offer several advantages over zirconia crowns, such as reduced tooth preparation, shorter operating times, and the ability to modify the crowns. Moreover, they offer satisfactory aesthetics and are cost-effective [7].

Minimizing pathological damage to natural teeth due to friction between restorative materials and opposing teeth is essential. Enamel wear is a progressive condition resulting from the continuous movement of the upper and lower teeth during mastication [8]. Additionally, the placement of restorations with wear properties different from the native tooth structure can exacerbate this natural process [9]. Excessive enamel wear can lead to clinical issues such as damage to occlusal surfaces, loss of occlusion’s vertical dimension, temporomandibular joint remodeling, reduced masticatory function, dentin hypersensitivity, tooth loss, and aesthetic impairments [10].

Assessing the color stability of restorative materials is crucial for determining treatment efficacy and patient satisfaction. The dynamic environment of the oral cavity, characterized by microorganisms, saliva, and the regular intake of pigmented foods and beverages, can affect the visual integrity of these materials. This poses a significant challenge for dental professionals [11].

This study aimed to assess the wear of crown materials and the opposing enamel of the recently introduced Bioflx crowns for deciduous teeth, compared to zirconia crowns. Additionally, the study aimed to examine the color stability of these crowns following thermodynamic aging and to evaluate the effect of beverages commonly consumed by children on the color stability of these crowns in a laboratory setting. The null hypothesis posited no significant differences between zirconia and Bioflx crowns regarding their wear resistance, impact on opposing enamel, or color stability after thermocycling and immersion in various solutions.

## Material and methods

This in vitro study was carried out at the Dental Biomaterials Department, Faculty of Dentistry, Suez Canal University, Egypt. Informed consent forms were signed by the parents or legal guardians of all children undergoing extractions at the Department of Pediatric Dentistry, Suez Canal University, granting permission for the use of their extracted teeth in the research. All of the performed procedures of the present study were authorized by the Research Ethics Committee (REC) of the Faculty of Dentistry at Suez Canal University with permission number 754/2023, conforming to the World Medical Association’s Helsinki Declaration (Version 2008).

The sample size was calculated using G*Power software version 3.1.9.2, following the methodology outlined by Faul et al. (2007) [12]. The software utilized an effect size convention (d) of 1.06, which is considered a large effect size. Based on the mean difference from previous studies, a statistical power of 95% was desired. The alpha (α) level was set at 0.05, and the beta (β) level at 0.05. Consequently, the required sample size (n) was determined to be at least 40, with 20 samples allocated to each of the two groups.

### Grouping

Forty-two primary mandibular right second molar crowns, size five, were used in this study (*n* = 42). Two crowns utilized for die production were excluded from the laboratory tests. The remaining crowns were divided into two groups: the zirconia group (*n* = 20, NuSmile, U.S.A. crowns) and the Bioflx group (*n* = 20, NuSmile, U.S.A. crowns). An equal number of mesiopalatal cusps from primary maxillary second molars were used as antagonists (*n* = 40).

### Die fabrication

Initially, two acrylic dies were fabricated by filling one crown of each type with self-cured acrylic resin (Acrostone, Egypt) and allowing it to be set for an hour. The crowns and dies were then assembled to ensure proper alignment. Visible undercuts in the dies were eliminated with a finishing bur. The dies were subsequently placed into polyvinyl chloride tubes filled with the same cold-cure acrylic resin to create a die resin base, which was also allowed to set for one hour. Two negative master silicon impressions (Tropicalgin, Zhermack S.p.A., Italy) were obtained from the two types of crown dies and were allowed to set for an hour. Twenty accurate acrylic dies for each crown type were fabricated and left to set for 24 h [13, 14].

### Crowns cementation

Self-cured glass ionomer cement (Riva, SDI, Australia) was used to cement the forty crowns onto their acrylic dies. The cement mixture was prepared according to the manufacturer’s instructions. The crowns were properly placed on the dies and allowed to be set for ten minutes under standard pressure. A specifically designed holding device was used to apply a consistent pressure of three kilograms on the crowns during the cementation procedure. Excess cement was eliminated with a dental explorer. The crowns were then allowed to undergo final setting for 24 h [15].

### Antagonist enamel preparation

An equal number of mesiopalatal cusps (*n* = 40) from primary maxillary second molars, which are naturally lost during the shedding process, were utilized as antagonists. Teeth exhibiting significant wear, fractures, cavities, restorations, or hypoplasia were excluded. The selected teeth were preserved in a solution of 0.04% thymol at a temperature of 4° C until needed for the experiment. Subsequently, the prepared samples were placed into molds using acrylic resin (Acrostone, Egypt) [16].

### Thermo-dynamic cycling

**Fig. 1:**
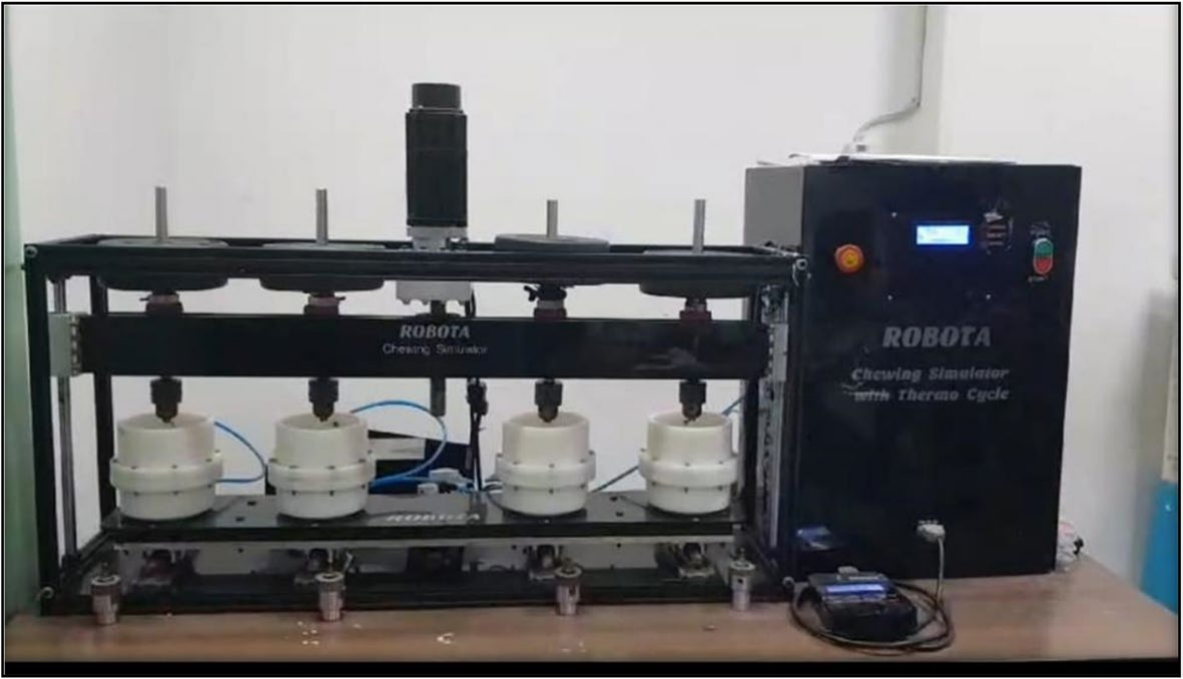
Chewing simulator (ROBOTA)

Thermodynamic Cycling: The crowns underwent 5,000 thermal cycles, with temperatures varying from 5 °C to 55 °C, using a Robota automated thermal cycler (BILGE, Turkey) [17]. Each cycle comprised a dwell time of 25 s and a lag time of 10 s, replicating six months of oral cavity conditions. After thermal cycling, an occlusal load of 50 N was applied 75,000 times at a frequency of 1.6 Hz utilizing a chewing simulator (Robota, model ACH-09075DCT, AD-Tech Technology Co., Ltd., Germany) [18, 19]. The chewing simulator consisted of four chambers, each equipped with an upper chuck for holding the mesiopalatal cusps of the maxillary second molars as antagonist specimens along with a lower Teflon holder for securing the crown specimens (Fig. 1).

### Methods of assessment

#### Wear testing

Optical methods were utilized to assess the volumetric changes (μm³) and the average roughness height (Ra) of the zirconia and Bioflx crowns, as well as their opposing antagonists. Before undergoing thermodynamic cycling (artificial aging), all specimens were initially scanned optically to obtain baseline measurements. Another optical scan was performed after the artificial aging process to obtain post-aging measurements [20, 21].

The specimens were imaged using a USB digital microscope (Guangdong, U500X Capture Digital Microscope, China) equipped with an integrated camera in order to provide a quantitative description of surface topography. The microscope was connected to a computer and maintained at a magnification of 120X. The images were analyzed using WSxM software, a specialized tool for scanning probe microscopy and nanotechnology applications [22]. The software expressed all measurements in pixels, including sizes, limits, frames, and parameters.

A three-dimensional profile of each specimen’s surface was generated. Three-dimensional images were obtained for each specimen within a rectangular area measuring 10 µm × 10 µm [23]. The WSxM software was used to calculate the volumetric changes (μm³) and the average roughness heights (Ra), expressed in micrometers (μm). According to established standards [24], these measurements serve as reliable indicators of the surface changes caused by wear.

#### Color- stability testing

The color changes in zirconia, and Bioflx crowns were assessed utilizing the L*, a*, b* (LAB) color scale developed by the International Commission on Illumination (CIE). This scale quantitatively measures color using three coordinates: L* (brightness or lightness), where black has an L* value of 0 and white has an L* value of 100; a* (hue), with red represented by positive a* values and green by negative a* values; and b* (chroma), with yellow represented by positive b* values and blue by negative b* values.

A dental spectrophotometer (X-Rite, model RM200QC, Neu-Isenburg, Germany) was used to measure the L*, a*, and b* color coordinates. The samples were positioned at the center of the spectrophotometer's measuring head using a white Teflon holder, ensuring that the probe tip was placed perpendicular to the center portion of the buccal surface of the crowns. For each sample, three distinct measurements were conducted, and the average values of L*, a*, and b* were calculated [25].

o **First Measurement (Baseline color values):** The first measurement (baseline) for the forty crowns was recorded immediately after cementation and before thermal aging.
o **Second Measurement (After Thermal Aging):** After the baseline color measurements, the samples underwent thermal aging for 5,000 cycles, simulating a clinical period of six months. The second color measurements were then taken using the same spectrophotometer. The measurements were conducted under identical conditions, by the same operator, and following the same procedures as the baseline measurements. The color change (ΔE*) between the two-color positions (first and second measurements) was calculated using the three-dimensional L*a*b* color space:
  $$\triangle{\mathrm E}_{\mathrm{CIELAB}}=\left(\triangle\mathrm L^{\ast2}+\triangle\mathrm a^{\ast2}+\triangle\mathrm b^{\ast2}\right)^\frac12$$

Where: L = * lightness (0–100), change the color of the axis (red/green) and b = * color variation axis (yellow/blue).

- **Third Measurement (After Immersion in Different Solutions):**

The samples from both groups (zirconia and Bioflx crowns) were further subdivided into four subgroups (*n* = 5) based on the immersion media (water, milk chocolate, orange juice, and Pepsi) [26]. The samples were immersed in 20 ml of each immersion medium and placed in an incubator at 37 °C (PS, Advanced Technology, Cairo, Egypt). The solutions were refreshed daily.

Following two weeks, the samples underwent a five-minute rinse with distilled water. The third measurement was conducted with the same spectrophotometer, under identical conditions, by the same operator, and adhering to the same protocols as the baseline measurements. The color difference (ΔE*) between the initial and final three-dimensional Lab* color space measurements was calculated.

### Statistical analysis

**Fig. 2:**
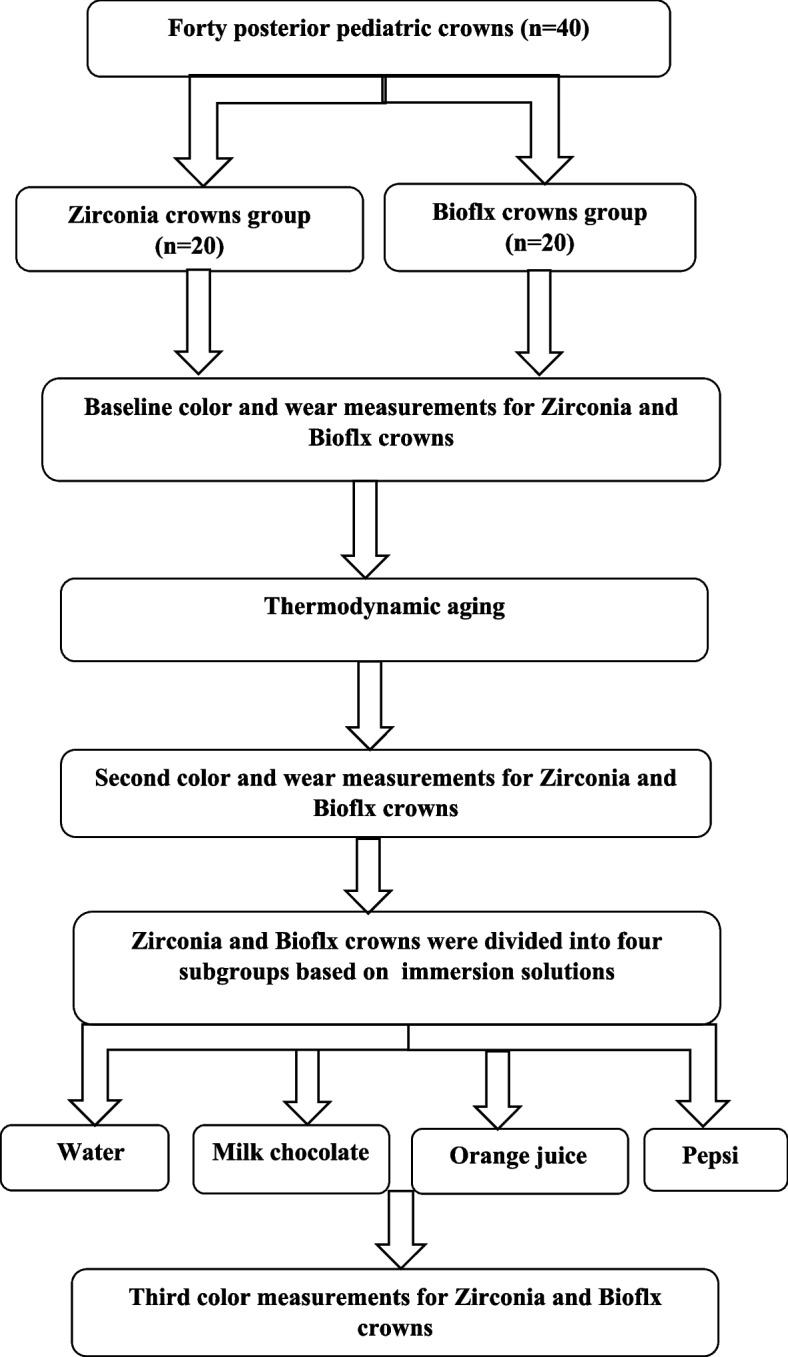
Schematic representation of experimental study design

All data were calculated, tabulated, and statistically analyzed using the appropriate tests. A Shapiro–Wilk normality test was conducted to determine whether the samples followed a normal distribution. Descriptive statistics were presented as the mean plus the standard deviation (SD). Paired sample t-tests were used to compare pre- and post-evaluation results within each group. Additionally, independent t-tests were employed to compare results between the groups. A *p*-value of ≤ 0.05 was considered statistically significant. All quantitative and statistical analyses were performed using SPSS software version 26.0 for Windows (Statistical Package for the Social Sciences, Armonk, New York: IBM Corporation).  The schematic representation of this experimental study was shown in (Fig. 2).

## Results

### Volume loss [µm^3^] and average roughness heights [µm] of natural antagonist enamel and crowns

**Fig. 3:**
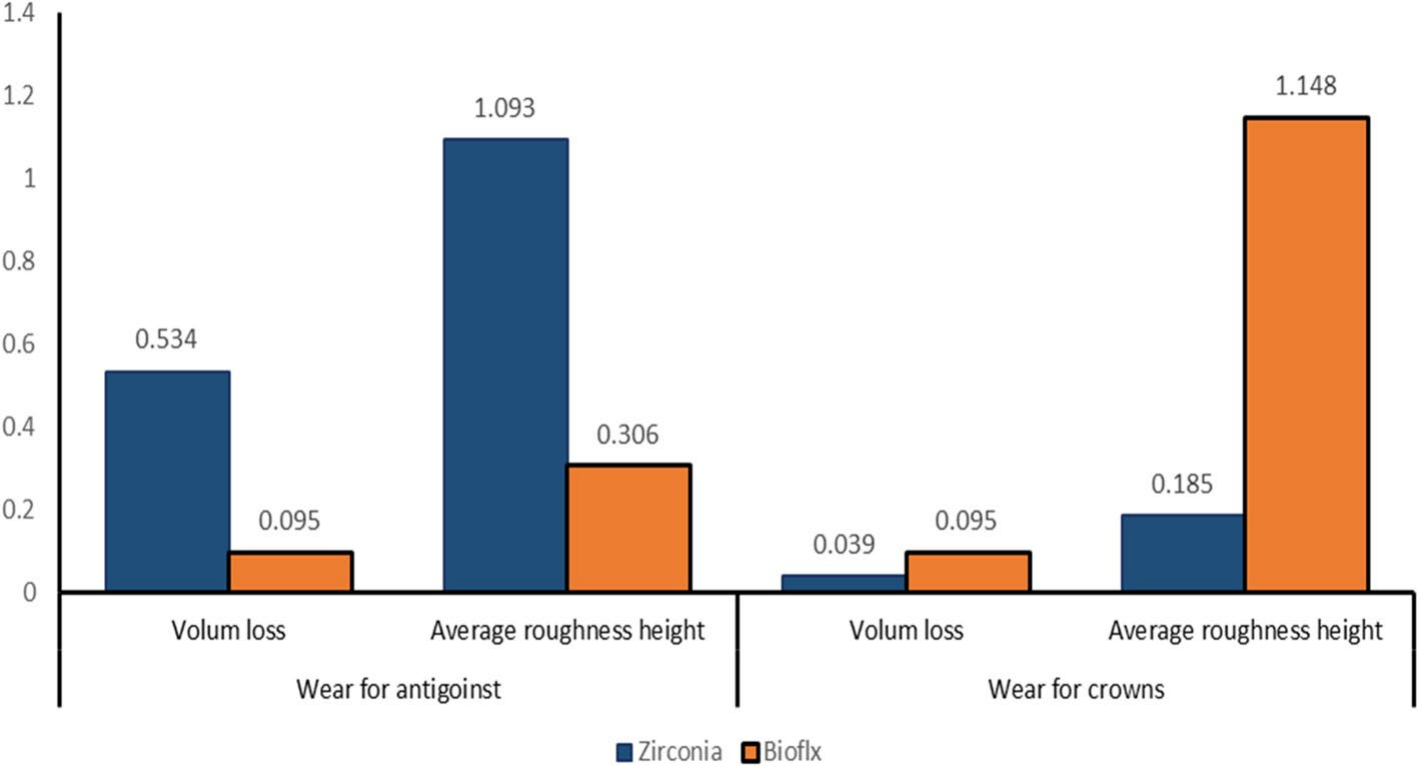
Antagonist enamel volume loss [µm.^3^] and average roughness heights [µm]

**Fig. 4:**
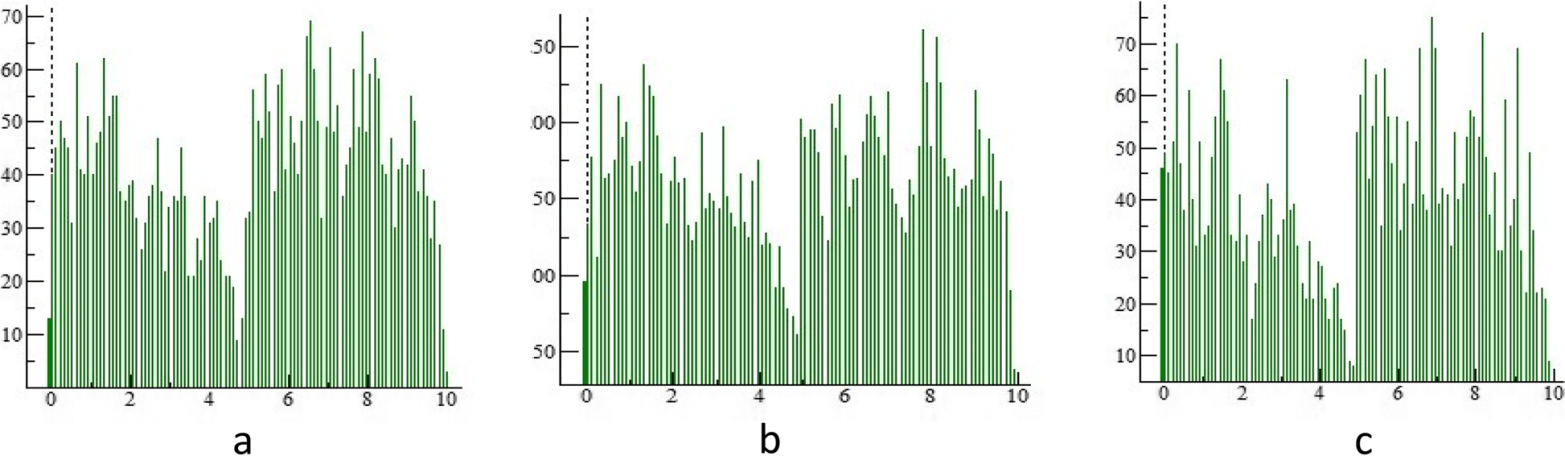
Histograms of antagonist enamel surfaces opposing different crowns before and after wear showing maximum and minimum worn areas. **a** Enamel surface before wear (baseline measurement). **b** Enamel surface opposing zirconia crown after wear. **c** Enamel surface opposing the Bioflx crown after wear

**Fig. 5:**
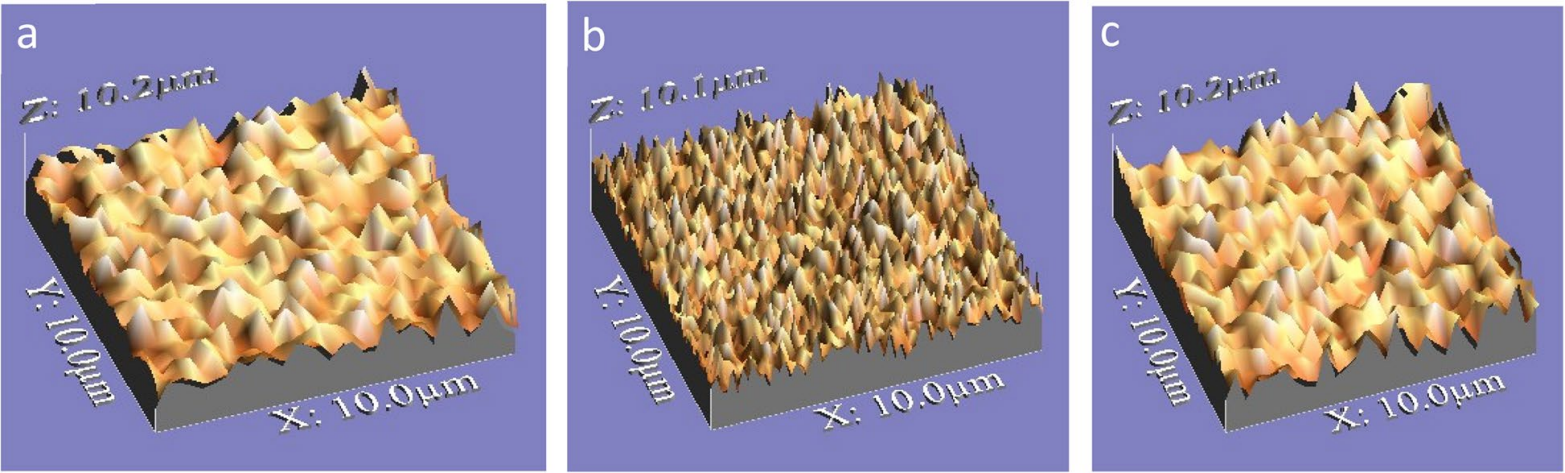
3D images of the antagonist enamel surface opposing different crowns before and after wear showing surface roughness. **a** Enamel surface before wear (baseline measurement). **b** The enamel surface opposing the zirconia crown after wear showing higher roughness. **c** The enamel surface opposing the Bioflx crown showing lower roughness

**Fig. 6:**
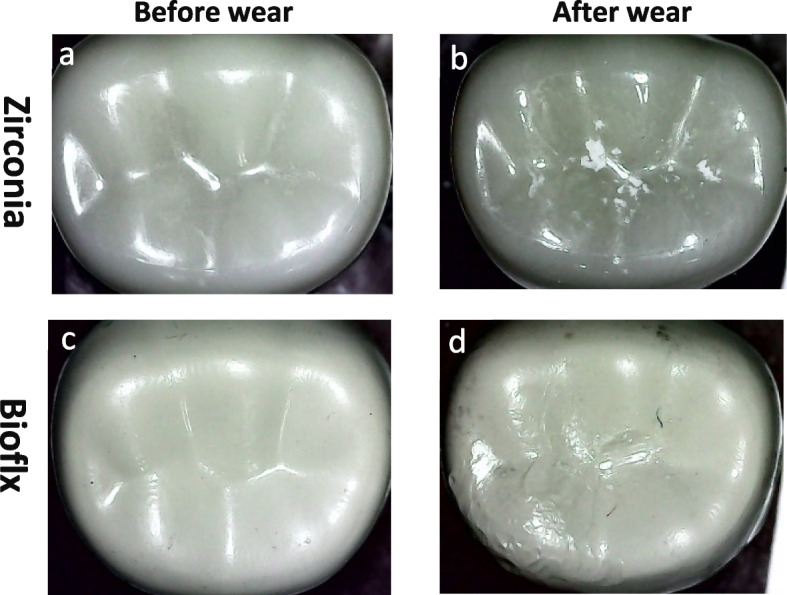
Zirconia and Bioflx crowns before and after wear (**a**) zirconia crown before wear. **b** zirconia crown after wear. **c** Bioflx crown before wear. **d** Bioflx crown after wear

**Fig. 7:**
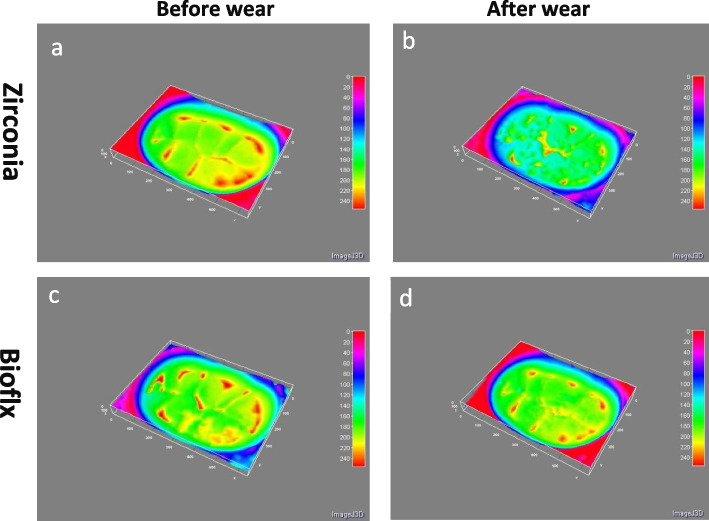
Head maps of zirconia and Bioflx crowns showing areas of indentation caused by wear. **a** zirconia crown surface before wear. **b** zirconia crown surface after wear. **c** Bioflx crown surface before wear. **d** Bioflx crown surface after wear

**Fig. 8:**
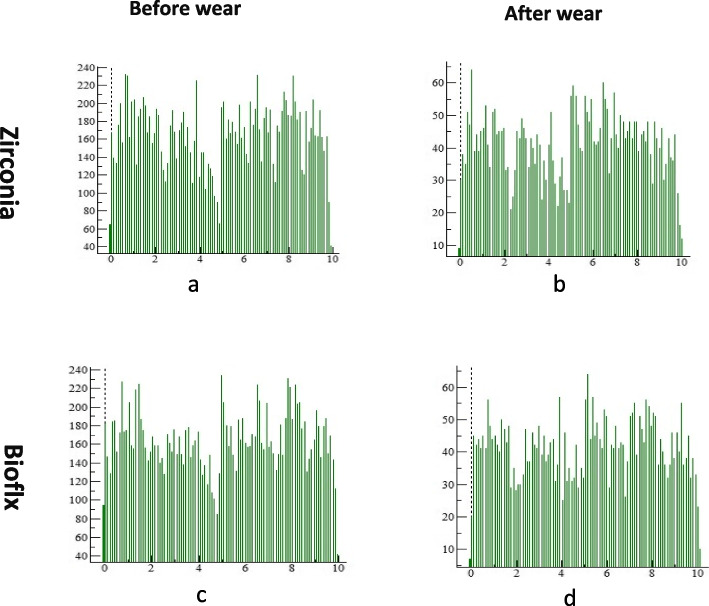
Histogram of zirconia and Bioflx surfaces before and after wear testing showing maximum and minimum worn areas. **a** zirconia surface before wear. **b** zirconia surface after wear. **c** Bioflx surface before wear. **d** Bioflx surface after wear

**Fig. 9:**
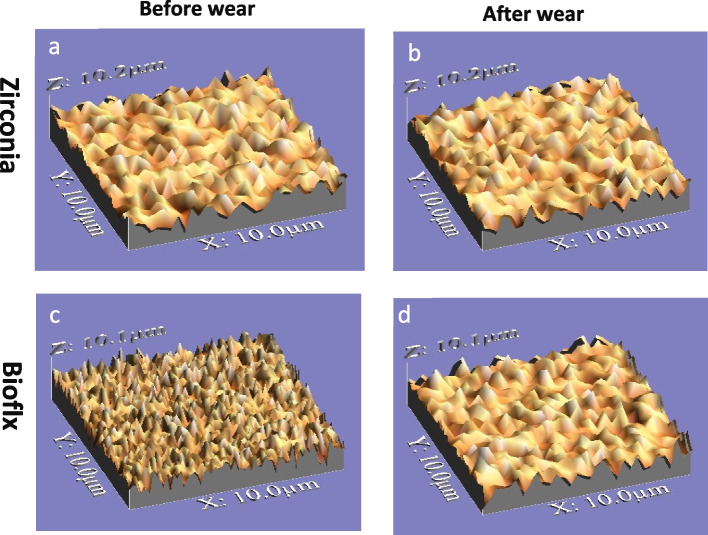
3D Images of zirconia and Bioflx crowns before and after wear showing surface roughness. **a** zirconia crown before wear. **b** zirconia crown after wear showing lower roughness. **c** Bioflx crown before wear. **d** Bioflx crown after wear showing higher roughness

Zirconia crowns exhibited a higher statistically significant difference regarding volume loss and average roughness heights of the antagonist enamel compared to Bioflx crowns (*P* = 0.021 and 0.001, respectively) (Table 1 and Figs. 3, 4, 5). Volume loss and average roughness heights for the Bioflx crowns indicated a higher statistically significant difference compared to zirconia crowns (*P* < 0.001) (Table 1 and Figs. 3 and 6, 7, 8 and 9).

**Table 1:** Volume loss [µm^3^] and average roughness heights [µm] of antagonist enamel and crowns

	**Antagonist**		**Crowns**		
	**Volume loss**	**Average roughness heights**	**Volume loss**		**Average roughness heights**
**Zirconia**	0.534 ± 0.163	1.093 ± 0.655		0.039 ± 0.046	0.185 ± 0.17
**Bioflx**	0.095 ± 0.091	0.306 ± 0.289	0.095 ± 0.091		1.148 ± 0.832
**Independent T test**	4.52	3.807	5.373		4.865
***P* value**	0.021*	0.001^a^	< 0.001*		< 0.001^a^

^a^Means significant difference

### Color ΔE

**Fig. 10:**
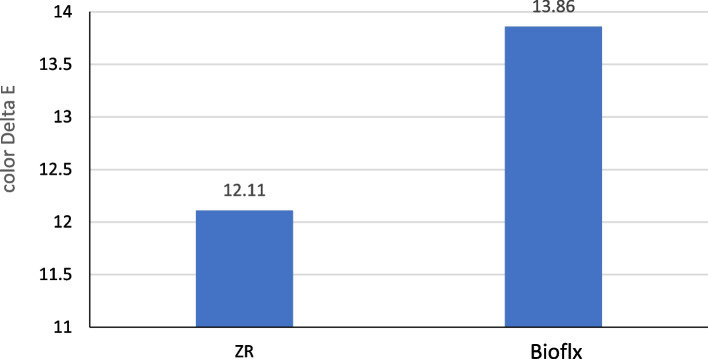
Colour ΔE of crowns before and after thermocycling aging

Color changes before and after thermocycling aging indicate no significant differences between the zirconia and Bioflx crowns (*P* = 0.472). However, the Bioflx crowns exhibited slightly high mean value (Table 2 and Fig. 10).

**Table 2:** Color ∆L, ∆A, ∆B, and ΔE of crowns before and after thermocycling aging

	**∆L**	**∆A**	**∆B**	**∆E**
Zirconia	5.056 ± 3.161	−2.200 ± 1.010	−0.344 ± 2.959	12.106 ± 6.216
Bioflx	12.267 ± 4.834	−1.244 ± 2.979	1.211 ± 0.065	13.864 ± 3.480
Independent T-test	−1.778	−0.575	−0.647	0.741
*P* value	${0.103}^{ns}$	${0.574}^{ns}$	${0.528}^{ns}$	${0.472}^{ns}$

**Fig. 11:**
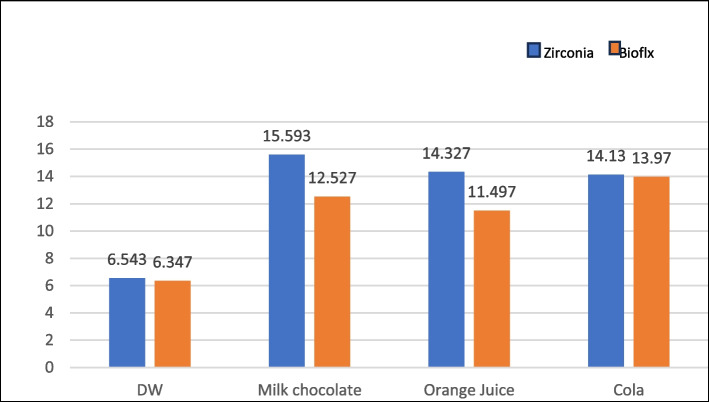
Color ΔE of crowns after immersion in different solutions

Color Changes after immersion in different solutions demonstrated no significant differences between the zirconia and Bioflx crowns. Additionally, no significant differences were observed in color change across the different solutions within each group (Table 3 and Fig. 11).

**Table 3:** Color ∆L, ∆A, ∆B, and ΔE of crowns after immersion in different solutions

	**Water**				**Milk chocolate**				**Orange Juice**			
	**∆L**	**∆A**	**∆B**	**∆E**	**∆L**	**∆A**	**∆B**	**∆E**	**∆L**	**∆A**	**∆B**	**∆E**
**Zirconia**	−1.800 ± 1.356	1.467 ± 2.871	−2.217 ± 1.000	6.545 ± 2.041	12.567 ± 2.892	−2.533 ± 3.113	8.633 ± 2.706	15.593 ± 4.353	−8.567 ± 1.007	1.667 ± 2.542	0.600 ± 1.401	14.327 ± 4.038
**Bioflx**	3.183 ± 0.825	4.317 ± 0.431	3.243 ± 0.615	6.347 ± 0.898	12.267 ± 0.473	0.100 ± 0.553	0.500 ± 1.735	12.527 ± 0.704	10.2667 ± 2.297	0.733 ± 1.436	2.167 ± 1.421	11.495 ± 7.357
**Independent sa sample** **T-test**	−1.593	−1.700	−2.337	0.153	0.177	−1.133	4.382	1.205	−3.004	0.378	−0.215	0.584
**P values**	0.186^*ns*^	0.164^*ns*^	0.139^*ns*^	0.886^*ns*^	0. 868^*ns*^	0.321^*ns*^	0.012^*^	0.295^*ns*^	0.040^*^	0.724^*ns*^	0.840^*ns*^	0.590^*ns*^

	**Cola**				**F test**	**P value**
	**∆L**	**∆A**	**∆B**	**∆E**		
**Zirconia**	−11.300 ± 3.751	0.300 ± 2.947	−3.750 ± 1.125	14.130 ± 4.404	3.466	0.071^*ns*^
**Bioflx**	12.500 ± 3.9837	−1.100 ± 2.078	3.950 ± 2.070	13.970 ± 1.888	2.240	0.161^*ns*^
**Independent sa sample** **T-test**	−7.534	.672	−1.335	0.058		
P** values**	0.002^*^	0.538^*ns*^	0.253^*ns*^	0.957^*ns*^		

^*^means significant difference

## Discussion

Bioflx crowns are a revolutionary development in pediatric dentistry, combining flexibility and adaptability to ensure an active fit to the tooth. They demonstrate significant potential in terms of aesthetics and conservative tooth preparation [6, 27].

The current study utilized the average roughness height (Ra) value to measure wear, as it is the most commonly used parameter for roughness determination. Ra facilitates comparability with other studies, is simple to define and measure, and provides a comprehensive general description of height variations [28]. Another indicator that is regarded as more sensitive is volumetric loss, which displays linear variations over time [29].

Ongoing research debates whether the enamel surface of natural teeth used as antagonists should be standardized for use in wear tests [30]. Krejci et al. [31] suggested that non-standardized enamel cusps are the most appropriate antagonists as standardization eliminates the prismatic enamel layer. Bolaca & Erdogan [16] also noted that standardized and non-standardized enamel surfaces have different wear properties. Therefore, this study used non-standardized enamel surfaces of the antagonist primary teeth.

The current study's findings demonstrated a statistically significant difference in wear between zirconia and Bioflx crowns, specifically regarding the volume loss and average roughness heights [µm] of the antagonist enamel. Zirconia crowns exhibited a higher degree of wear than Bioflx crowns. This increased wear was attributed to the insufficient ductility of ceramic materials, leading to fractures or chipping. These chips act as abrasives on the ceramic surface, increasing wear rates [32]. Fatigue wear contributes to the formation of microscopic reticular cracks, numerous small enamel chips, and pit-like structures [33]. This finding is consistent with Aly et al. [34], who demonstrated that primary teeth experienced more severe wear due to mechanical mismatching between zirconia crowns and natural enamel. Zirconia crowns possess superior mechanical properties, including a flexural strength greater than 1,000 MPa, an elastic modulus of 210 GPa, and a hardness of 10 GPa. These values are significantly higher than human enamel, which has a flexural strength of 280 GPa, an elastic modulus of 94 GPa, and a hardness of 3.2 Gpa [35, 36]. These findings were corroborated by other studies [37, 38]. including Tang et al. [39]. They concluded that localized contact generates substantial compressive stress on the enamel and leads to enamel fatigue when repeated over time. Despite the wear associated with zirconia crowns, it was considered clinically acceptable [40]. Conversely, Wang Lin et al. [33] reported no significant difference in the amount of enamel wear when comparing zirconia crowns to composite resin crowns. Similarly, Choi et al. [32] and Bhatt et al. [41] found minimal differences in the durability of zirconia crowns versus stainless steel crowns concerning the wear of deciduous teeth.

The finishing and polishing methods for zirconia restorations are crucial and can substantially affect the wear outcomes reported in studies. Improperly finished or polished zirconia may exhibit abrasive properties, increasing wear on the antagonist teeth [42]. Without precise information regarding the finishing and polishing of zirconia, clinicians may mistakenly believe that all zirconia restorations substantially elevate the risk of wear on opposing teeth. However, this risk can be mitigated through proper finishing methods. Janyavula et al. [43] concluded that polished zirconia is wear-friendly to the opposing tooth, but glazed zirconia causes more material and antagonist wear than polished zirconia.

Regarding the wear resistance of the tested crowns, the results showed a significant difference in volume loss and average roughness heights between zirconia and Bioflx crowns. Bioflx crowns exhibited a higher mean value of wear compared to zirconia crowns. These differences can be primarily attributed to the distinct material properties, such as variations in hardness and elastic moduli. The wear behavior of ceramics is also influenced by factors including microstructure, porosity, surface roughness, crystal size, and environmental conditions [44]. The inclusion of silicate and zirconia clusters using innovative nanotechnology is anticipated to improve the material’s stability and resistance to wear, according to the manufacturer. In Bioflx crowns, wear occurs when particles become displaced from the polymer matrix due to the impact of the antagonist. The size, hardness, and volume percentage of the fillers used in the material significantly impact its wear characteristics [45]. These findings align with those of Patil et al., [5] who observed no signs of opposing tooth wear with Bioflx crowns in primary molars after a 12-month follow-up. They also reported partial occlusal surface wear of Bioflx crowns without exposing the underlying tooth surface. Additionally, the self-adaptive properties of these crowns create dimples in high occlusal areas, thereby preventing further wear.

The measurements of reflective surfaces can be affected by the material's inherent color and the lighting conditions present during the evaluation. In this investigation, a standard illuminant was used against a white background to ensure reliable and consistent measurements [46]. In this study, chocolate milk, orange juice, and Pepsi were selected as the immersion media because these beverages are among the most frequently consumed by children and teenagers in the Middle East. Specimens were stored in these staining solutions at 37ºC to simulate the normal oral cavity temperature [46, 47].

The current study found no significant differences in color stability between zirconia and Bioflx crowns regarding color change before and after thermocycling aging and immersion in different solutions. The color change (ΔE) values exceeded the acceptability and perceptibility thresholds [48], with higher values indicating a more noticeable color difference to the human eye [49]. Artificial colorants and citric acid contribute to the discoloration of teeth and restorations. Additionally, the acidic pH of these soft beverages is a factor that contributes to the yellowing of dental restorations. Therefore, the increase in ΔE values after immersion in soft drinks can be attributed to these factors [50]. The staining potential of various beverages and solutions varies significantly due to differences in composition and inherent properties [51]. The study’s findings demonstrated that the hue of the materials changed after immersion in chocolate milk, orange juice, and cola compared to color change in the water control group. The staining effect of chocolate milk can be linked to its organic constituents and lipid content [52]. The discoloration resulting from cola and orange juice can be attributed to their respective acidity levels, with cola and orange juice exhibiting pH levels of 3.0 and 4.2, respectively. The acidic pH of these drinks likely affected the structure of the crown materials tested [11, 46].

Numerous studies have examined the color stability and stain resistance of zirconia crowns over durations of 1 to 36 months after placement. Research indicates that zirconia crowns demonstrate significant color stability and resistance to staining [53, 54]. The surface characteristics of zirconia, particularly its high hardness, confer resistance to scratches, leading to a smooth, shiny, and polished finish. The low surface energy of zirconia crowns inhibits plaque and bacterial adherence, thereby preventing staining and color deterioration [55, 56]. Bioflx crowns, composed of a biocompatible hybrid resin polymer, resolve the issues associated with durability, color stability, and flexibility frequently encountered in composite crowns reinforced with fiberglass. Patil et al. [5] found that Bioflx crowns exhibited stain resistance, with no visible discoloration noted after one year of intraoral use. However, there is a lack of extensive studies evaluating the characteristics of Bioflx crowns, as well as their impact on clinical outcomes and parental satisfaction.

The null hypothesis regarding wear resistance was rejected, as significant differences were observed between zirconia and Bioflx crowns. However, the null hypothesis regarding color stability was accepted, as no significant differences were found between zirconia and Bioflx crowns.

This study has some limitations. First, the limited literature on Bioflx crowns, being a newer technology, restricts the depth of analysis and comparison with established materials. Second, the study focused on two-body wear, which doesn’t fully capture real-world chewing conditions that often involve three-body wear (with food particles). Lastly, it didn't consider other factors like saliva and food debris, which can influence wear in actual use. Future research should address these gaps by exploring both wear types and more complex real-life factors for a better understanding of Bioflx crowns' performance.

Clinical in vivo studies are recommended to assess the long-term durability and performance of Bioflx crowns in primary molars.

## Conclusions

- Zirconia crowns cause significantly more wear on opposing natural teeth compared to Bioflx crowns.
- Bioflx crowns demonstrate a superior average wear rate relative to zirconia crowns.
- In terms of color changes, no significant differences are noted between zirconia and Bioflx crowns following aging and immersion in different solutions.

## Data Availability

The corresponding author is available to provide the datasets used and/or generated in this study upon request
